# Development and validation of clinical performance assessment in simulated medical emergencies: an observational study

**DOI:** 10.1186/s12873-015-0066-x

**Published:** 2016-01-15

**Authors:** Aysen Erdogan, Yue Dong, Xiaomei Chen, Christopher Schmickl, Ronaldo A. Sevilla Berrios, Lisbeth Y. Garcia Arguello, Rahul Kashyap, Oguz Kilickaya, Brian Pickering, Ognjen Gajic, John C. O’Horo

**Affiliations:** Department of Medicine, Mayo Clinic, Rochester, MN USA; Department of Anesthesiology, Mayo Clinic, Rochester, MN USA; Department of Anesthesiology and Reanimation, Suleyman Demirel University, Isparta, Turkey; METRIC group, Mayo Clinic, Rochester, MN USA; Department of Critical Care Medicine, Qilu Hospital of Shandong University, Shandong, China; Department of Internal Medicine, Boston University Medical Center, Boston, MA USA; Department of Anesthesiology and Reanimation, Gulhane Medical Faculty, Ankara, Turkey

## Abstract

**Background:**

Critical illness is a time-sensitive process which requires practitioners to process vast quantities of data and make decisions rapidly. We have developed a tool, the Checklist for Early Recognition and Treatment of Acute Illness (CERTAIN), aimed at enhancing care delivery in such situations. To determine the efficacy of CERTAIN and similar cognitive aids, we developed rubric for evaluating provider performance in a simulated medical resuscitation environments.

**Methods:**

We recruited 18 clinicians with current valid ACLS certification for evaluation in three simulated medical scenarios designed to mimic typical medical decompensation events routinely experienced in clinical care. Subjects were stratified as experienced or novice based on prior critical care training. A checklist of critical actions was designed using face validity for each scenario to evaluate task completion and performance. Simulation sessions were video recorded and scored by two independent raters. Construct validity was assessed under the assumption that experienced clinicians should perform better than novice clinicians on each task. Reliability was assessed as percentage agreement, kappa statistics and Bland-Altman plots as appropriate.

**Results:**

Eleven experts and seven novices completed evaluation. The overall agreement on common checklist item completion was 84.8 %. The overall model achieved face validity and was consistent with our construct, with experienced clinicians trending towards better performance compared to novices for accuracy and speed of task completion.

**Conclusions:**

A standardized video assessment tool has potential to provide a valid and reliable method to assess 12 performances of clinicians facing simulated medical emergencies.

**Electronic supplementary material:**

The online version of this article (doi:10.1186/s12873-015-0066-x) contains supplementary material, which is available to authorized users.

## Background

Critical illness is a time-sensitive process requiring practitioners to process vast quantities of data and make rapid decisions. Our group developed an electronic checklist and cognitive aid, CERTAIN (the Checklist for Early Recognition and Treatment of Acute Illness) based on a survey of provider needs [1]. The software is designed to assist practitioners with point-of-care decision support for the acutely decompensating patient. It offers an innovative interface to track patient information, resuscitation actions and reference tools for common resuscitation scenarios.

Before clinical implementation of this new tool, we needed a rubric to evaluate the feasibility and usability of the CERTAIN software. Formal evaluation of performance of new technology in clinical environment is difficult, and thus we sought to accomplish this in a simulated clinical environment. Simulation based assessment has been used for measure team performance, communicational skill in various healthcare setting of trauma, anesthesia and operation room, and emergency department [2–6]. However, few validated tools currently exist specifically designed to evaluate individual provider technical performance in resuscitation scenarios. The Checklist for Early Recognition and Treatment of Acute Illness (CERTAIN) has been recently developed with intent to facilitate structured, disciplined approach to medical and surgical emergencies [1, 7–9]. In order to test the efficacy of this and other acute care decision aids, we sought to develop a rubric for evaluating provider performance in a simulated medical resuscitation environment allowing for reliable grading of performance of critical care tasks and effective discrimination of experienced versus novice clinicians.

## Methods

This was a prospective observational study, where all participants went through a simulated scenario unaided. Human subject approval was sought and obtained from the Mayo Clinic Institutional Review Board (Approval #13-003927). All participants verbally consented to being observed and recorded. All subject recruitment and observations were performed between 9/2013 and 5/2014.

Study participants consisted of medical students, residents, visiting clinicians, and critical care fellows. At minimum, subjects were required to have had previously been certified in Acute Cardiac Life Support (ACLS). None of the subjects were provided with additional team or resuscitation training at start, such as Team STEPPS or Fundamentals of Critical Care Support. Subjects were recruited from the Mayo Clinic in Rochester, MN, and its associated hospitals and medical school. E-mails, flyers, and contact with residency programs and fellowships were used to recruit subjects. A chance to win an iPad mini was offered to eligible clinicians as an incentive to participate. We targeted an enrolment of 30 based on availability of simulation center resources and anticipated time available for clinicians to participate in research off of the main clinical campus.

The study was performed in the Mayo Clinic Multidisciplinary Simulation Center, a facility which regularly performs research and education using high-fidelity simulation tools. The simulation rooms are each equipped with several cameras to allow for recording of clinician actions from different points of view for later viewing and assessment (B-Line Medical, Washington, DC), as well as a mannequin (Laerdal Medical®, Stavanger, Norway) procedure cart, medications, and other medical supplies [10].

With input from simulation center personnel, we developed three scenarios to assess provider’s medical management of emergencies frequently encountered in general practice requiring intensive care (ICU) hospitalization: 1) Low blood pressure due to sepsis; 2) shortness of breath due to pneumonia; 3) chest pressure and palpitation due to acute coronary syndromes (ACS). All cases were limited to 10 min, intended to simulate approximately 30 min of “real time” interventions in a compressed manner. There were two study personnel acting as assistants to help participants during the testing scenarios.

**Fig. 1:**
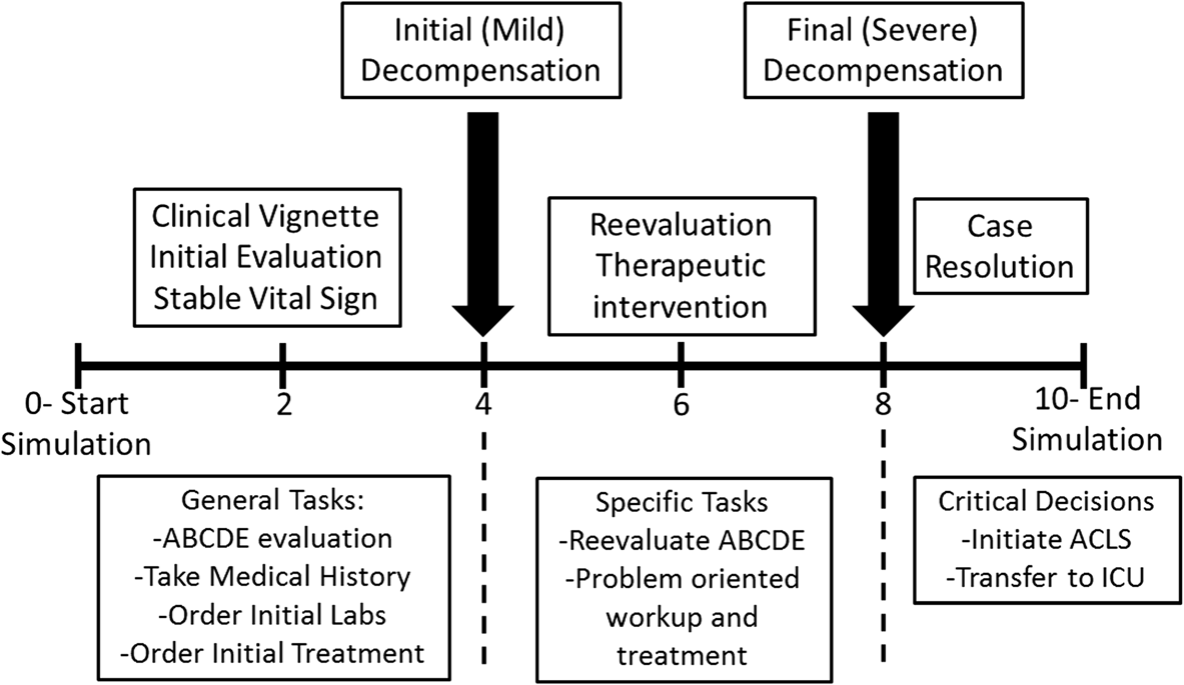
General timeline of a simulation

Before the case started, volunteers received a standard orientation to the simulation center and the simulation environment capabilities. Each case started with a clinical vignette consisting of a brief description of the presenting problem, including patient age, gender, origin, arrival transportation, and chief complaint. The scenario then initiated and progressed through three stages. In the first four minutes, the patient remained stable for initial evaluation, history, and workup. At the end of this stage, the patient decompensated with changes in vital signs or a new complaint. This was followed by a more drastic decompensation at 8 min, suggesting the need for critical care disposition decisions before case resolution (See Fig. 1). A full script for one of our scenarios is in Additional file 1.

Based on best practice models, we designed a checklist of critical actions that should be undertaken in each scenario to evaluate the participant’s performance [11, 12]. This initial checklist was based on face validity of content when reviewed by critical care experts, with the intent that our construct and reliability assessments would refine it further. The metrics were developed iteratively by four experts: two critical care anesthesiologist, one pulmonary critical care specialist and one emergency medicine specialist. Modified Delphi process was used to include examples of both general and scenario –specific components. Each checklist included items such as 1) evaluating resuscitation preference code status; 2) primary assessment of airway, breathing, and circulation, 3) scenario-specific interventions for every patient (Table 1). Assessments of technical procedural skills (e.g., central line placement, intubation, CPR, etc.) were not included. Each case also had scenario specific tasks, which were similarly measured (Additional file 1).

**Table 1:** Definitions for items common to all cases. Vital signs were provided by a monitor that gave pulse oximetry, heart rate, respiratory rate and blood pressure. As the monitor does not provide temperature, this was scored as a separate task

Item	Definition for task completion
Resuscitation Code status	Any discussion with the patient or nurse about whether the patient wants CPR and/or intubation
Primary assessment: Airway	Explicitly addresses any of the following: Airway compromise, stridor, wheezing; Alternatively may say something like: “adequate airway”, etc.
Primary assessment: Breathing	Explicitly addresses any of the following: Poor air entry, Crackles, Work of Breathing; Alternatively may say something like: “apparently no breathing problems”, or ask for/do lung auscultation etc.
Primary assessment: Cardiac	Explicitly addresses any of the following: ECG monitor, pulse status, mottling; Alternatively may ask for “cardiac monitoring”, or say something such as: “intact circulation”, etc.
Primary assessment: Disability	Explicitly addresses any of the following): Level of consciousness (AVPU: Awake, verbal responsive, pain responsive, unresponsive), seizures, focal deficits; Alternatively may say something like: “apparently awake and oriented/ unresponsive”, etc.
Primary assessment: Exposure	Explicitly addresses any of the following): Abdominal distension, overt bleeding, skin abnormalities evaluation (rash, wound, Jaundice, Sc. emphysema, edema)
Check vital signs	Asks for vital signs
Check temperature	Asks for temperature; Alternatively may ask for fever or if the patient feels hot/cold etc.
Review past medical history	Asking for past medical history/previous diagnoses to the mannequin or nursing personnel
Review medications	Asking for home medications to the mannequin or nursing personnel
Review allergies	Asking for known drug allergies to the mannequin or nursing personnel
Review differential diagnosis	Considers and verbalizes at least one alternative diagnosis different from the (apparent) working hypothesis
Order labs	Ordering any lab tests (including point-of-care labs)
Order oxygen	Verbalizes consideration of the need of or ordering supplemental oxygen (any FiO2, any device)

All simulation sessions were recorded and stored by secured AV system (B-Line Medical, Washington DC.). Two independent physician raters viewed each recording, and rated items as performed or not performed, and recorded the time at which the task was completed. The raters practiced scoring on training videos and definitions refined and summarized in a standard operating procedure (SOP) to achieve better agreement. Ultimately, any disagreements between raters were adjudicated by a third critical care physician.

Reliability was assessed using Cohen’s Kappa coefficient statistics as well as percentage agreement between the two reviewers based on initial (non-adjudicated) impressions. Both kappa and percent agreement was calculated for the common items, as the N for this set was larger. For the case-specific items, only percentage agreement was used given the small number of cases potentially leading to paradoxically lower Kappas in cases of high agreement [13]. We decided *a priori* that 60 % agreement would be considered adequate for our purposes.

We also assessed the reliability of the time completion assessments for each item using a Bland-Altman plot, as well as creating an aggregate plot for overall timing agreement for all items. Any item that did not attain the reliability threshold both on completion and timing was excluded from further analysis.

Clinicians were stratified into two categories; experienced and novice ICU clinicians. Experienced clinicians were defined as having at a minimum of 6 months of formal critical care training and novice ICU clinicians as those who spend more time outside of the ICU (e.g. medical students, residents and hospitalists). In our construct, experienced clinicians should perform as well or better than novice clinicians if the tool accurately assesses clinicians’ critical care skills. Given the small N, we did not expect any of our measures to necessarily reach statistical significance, and instead looked at the overall trend.

Time to completion was not used as a major determinant of discriminative validity given individual practice variations in the order in which items are addressed and treated, and the small sample size.

## Results

### Study participants characteristics

Twenty-five clinicians with various levels of critical care experience and current, valid ACLS certification were enrolled over a nine month study period. Six participants dropped out before the formal simulation testing due to scheduling difficulties, and one was excluded because of a mistake in how the scenario was recorded, leaving a total of 18 participants for evaluation. Eleven participants met our definition of “expert,” and seven of “novice” critical care provider (Table 2).

**Table 2:** Professional characteristics of the participants*

Position	Participants (*N* = 18)	Years of since medical school graduation	Months of Critical care training
Critical Care Fellows	11	2.5	18 months (3) 6 months (6)
Medical Residents	2	2	0
Medical Students	3	0	0
Visiting Physicians	2	2	0

*all participants had valid and current ACLS training

### Reliability assessments

**Fig. 2:**
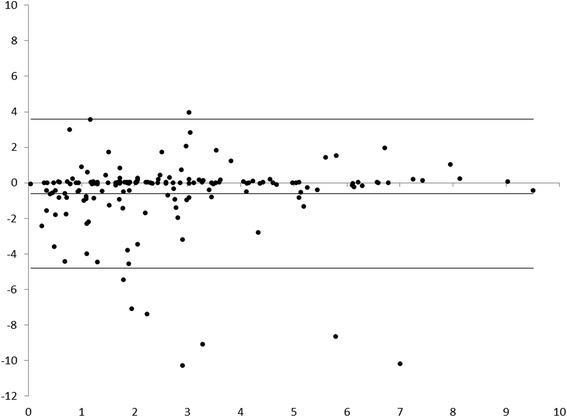
Plot of difference between reviewer 1 and 2 ratings for the time any given item is completed plotted against the mean of those two values. Evidence of bias is minimal, and reliability, with mean difference of −0.61 min, is satisfactory

With regards to completion of items, the common checklist tested well for reliability, with all but one item (airway assessment) meeting our 60 % cut off, and most items meeting or exceeding 80 %. (see Table 3). Overall, kappa scores were also adequate, with an overall rating of 0.61, and only airway (−0.21) and neurologic (0.12) having low kappa scores. Timing assessments were generally satisfactory, with minimal evidence of bias on the Bland-Altman plot. The mean difference between reviewer ratings was 0.61 min (Fig. 2). Individual items with higher variability were neurologic and circulation assessments, checking vitals, oxygen administration, and verbalizing the differential diagnosis.

**Table 3:** Common checklist items. Items marked with * were below our threshold for reliability, and were not included in the final rubric. Items with Kappa marked as “undefined” were performed in 100 % of cases

Item	% Overall Agreement/Kappa	Kappa
Discussed Code Status?	67 %	0.40
Assessed Airway?*	47 %	−0.21
Assessed Breathing?	100 %	Undefined
Assessed Circulation?*	100 %	Undefined
Assessed Disability? (Neurologic status)*	80 %	0.12
Assessed Skin/exposure?	80 %	0.56
Obtained vitals?*	100 %	Undefined
Obtained temperature?	80 %	0.66
Obtained past medical history?	100 %	1.00
Obtained medications?	93 %	0.78
Obtained allergies?	80 %	0.65
Obtained labs?	93 %	0.45
Administered oxygen?*	93 %	Undefined
Verbalized Differential Diagnosis?*	73 %	0.40
Pooled reliability:	84.8 %	0.61

In the case-specific assessments, data, case 1 had excellent agreement on task completion, but somewhat disagreement on timing of IV fluids boluses, vasopressor initiation, and intubation administration. Several items were uniformly not performed, making agreement on timing not calculable (Additional file 2).

Case 2 specific items similarly were generally good, though there was significant disagreement again on timing and administration of fluid boluses, identification of sinus tachycardia, and whether antibiotics and blood cultures were ordered. Timing agreement was, however, generally very good in this group (Additional file 2).

Case 3 specific items scored the lowest overall, with reasonable agreement on task completion, but discrepant timing (See Additional file 2).

### Discriminative validity

Experts outperformed less experienced clinicians in accuracy and timeliness of assessment in the majority of general and case-specific items. The only truly notable exceptions were temperature and exposure assessments in the common tasks, (see Tables 4 and 5) and thus we excluded these from our master rubric. Because of our small number involved in each individual case, our ability to evaluate individual items for discriminative validity was limited. However, the overall task rate completions for case specific items in all three cases were better with expert clinicians than novices, consistent with our construct (Additional file 2).

**Table 4:** Common checklist items: completion by experts vs. novices

Item	Completed by experts (*N* = 11)	Completed by novices (*N* = 7)
Discussed Code Status?	6 (55 %)	1 (14 %)
Assessed Airway?	6 (55 %)	4 (57 %)
Assessed Breathing?	11(100 %)	7(100 %)
Assessed Circulation?	11(100 %)	7(100 %)
Assessed Disability? (Neurologic status)	10(91 %)	4(57 %)
Assessed Skin/exposure?	4(36 %)	5(71 %)
Obtained vitals?	11(100 %)	7(100 %)
Obtained temperature?	5(45 %)	5(71 %)
Obtained past medical history?	8(73 %)	4(57 %)
Obtained medications?	7(64 %)	0(0 %)
Obtained allergies?	6(55 %)	2(29 %)
Obtained labs?	11(100 %)	6(86 %)
Administered oxygen?	11(100 %)	7(100 %)
Verbalized Differential Diagnosis?	9(82 %)	5(71 %)
Pooled reliability:	73.5 %	65.3 %

**Table 5:** Median time to completion for experts vs novices. Tasks with ** took experts longer than novices

Items	Expert time to completion (Minutes)	Novice time to completion (Minutes)
Code status	5.2	6.0
Breathing**	2.2	1.3
Exposure**	3.0	2.1
Temperature	4.1	2.1
PMH	1.9	1.9
Meds	2.1	N/A
Allergies**	3.7	2.7
Labs	2.7	3.2
Case 1 specific items		
Obtained cultures (any type)	2.6	6.6
Obtained blood cultures?	3.1	6.6
Antibiotics given?	3.9	7.2
Sedation performed	10.3	11.3
Case 2 specific items		
Identified wheezing?	1.9	1.9
Identified crackles?	2.0	2.8
Obtained cultures (any type)**	2.9	2.2
Chest X ray obtained?	2.7	3.4
Intubation preparation discussed?	5.4	5.7
Preoxygenation performed?	5.8	8.4
Sedation performed**	7.0	6.7

**Fig. 3:**
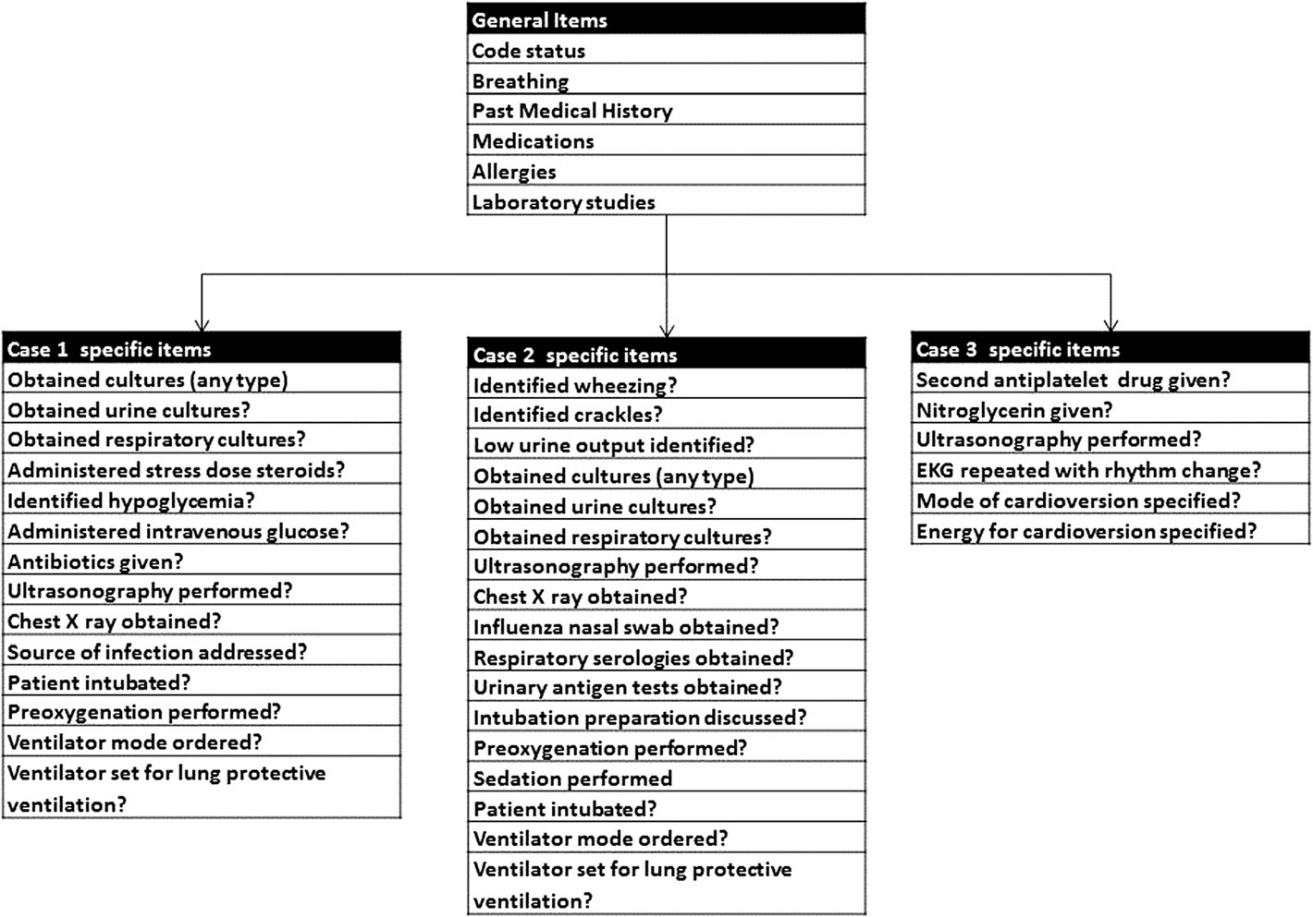
Final itels included in the rubric

Overall, experts took less time to address each task as well (Additional file 2), again, consistent with our construct. With exclusion of the items failing our threshold for reliability, and the common checklist items failing our construct, we generated our final rubric (Fig. 3).

## Discussion

In our observational study, six general assessment items and 37 case specific items were identified as both valid and reliable for assessing provider decision making performance in simulated medical emergencies. Several items that were tested and excluded (i.e. airway assessment) are undoubtedly important, but, like most simulation assessments, the goal of this study was not to be a comprehensive rating tool [14]. Rather, we sought to make a focused tool to allow usability testing of specific decision support models such as CERTAIN [15].

The Observational Skill Based Clinical Assessment Tool for Resuscitation (OSCAR) and Team Emergency Assessment Measure (TEAM) used similar development models to develop a rubric for assessing the non-technical skills (i.e. decision making) critical to leading resuscitation teams. Like our rubric, OSCAR and TEAM were developed based on face validity and content expertise, and optimized for inter-rater reliability. However, both were primarily targeted at evaluating teams rather than individuals, and OSCAR specifically evaluates communication skills [6, 16]. As such, these do not look for specific binary behaviors (i.e. “identifies hypoglycemia”), but rather rates the qualitative aspects of communication and its impact on team functioning. Similar tools have been developed for other settings, such as crew resource management, [16] anesthesia, [3] and surgery [17, 18] using similar methodologies.

A tool designed by Ottestad et al. [19] attempted to measure performance of simulated initial sepsis resuscitation. That study identified a series of desired behaviors and decision point similar to our case one checklist (e.g. fluid bolus yes/no, obtains central access, orders antibiotics, etc.) and general checklist (e.g. verbalizes differential diagnosis), but also included a subjective rating of communication, planning and leadership skills [19]. Inter-rater reliability was tested for the overall score and was quite good, but was reported across global dimensions (e.g. technical skills) as opposed to specific items. Another critical care construct was described by Boulet et al., [20] who made ten clinical scenarios with a pre-defined list of priorities the provider would be expected for each. Medical students and residents were tested in simulated medical emergencies. Their tool had high inter-rater reliability, and demonstrated a trend towards discriminatory validity with more time spent in critical care training being associated with better performance in two of the cases they developed [20].

Our tool differs from the majority of existing systems in two key ways. Firstly, we sought to make an evaluation tool that targets behaviors which differentiate clinicians who spend the majority of their time in critical care settings from others. Most simulation scoring systems in critical care environments to date were designed to provide formative or summative assessment of trainees. As such, most existing tools focus on nontechnical skills like teamwork and communication. We allowed our team leaders to assume leadership “best practices,” [21] such as egalitarian leadership [22, 23], closed-loop communication [24], and briefing/debriefing [25], but we did not require nor grade these behaviors. Our main interest was to develop a rubric to evaluate the impact of clinical decision support on resuscitation practices, and these types of behaviors fall outside of this realm. As such, this tool can also be used to evaluate if the design meet specific performance and/or stratification goals during the formative assessment stage. It will serve as usability benchmark for the future development [6, 26].

Secondly, our tool targets medical deterioration rather than operating room, anesthesia or cardiac arrest settings. Although some discuss medical resuscitation [19, 20], most are intended for settings other than medical wards and emergency rooms [2–5]. Most medical resuscitation simulations, like ACLS “megacodes,” assess perimortem assessment and treatments, and thus miss the opportunity to assess behaviors that can prevent cardiac arrest. Ours allows for insight into an area of care that allows for meaningful early interventions.

Our study was limited by the small number of participants, limiting statistical power. This was aggravated high dropout rate among participants. Six who enrolled were not able to complete their evaluation due to scheduling conflicts, comprising nearly a quarter of the study population. The reasons for this are not entirely clear, but may have reflected inappropriate incentives for participation, as well as the fact that the simulation center was only available during business hours, when many expert clinicians were working and novices had classes. When rating a clinical decision tool, availability of clinicians to provide time and input for real-world usability is often a rate limiting step, and is why there is so little research on validation of such tools.

The rating system was developed in a simulation center of a single tertiary care institution and validation in other simulation centers and scenarios is required. We also purposefully did not measure the procedural skills and non-technical skill of other team members as that is outside of the purview of clinical decision support tools, but these non-technical aspects are important as well. In our statistical analysis, we chose an arbitrary cutoff of 60 % agreement, which may not be perceived as adequate; fortunately, most of our metrics exceeded 80 %, so our tool still performed reliably. Several items were not performed by either expert or novice clinicians, which may indicated failures in our initial selection process. However, we were seeking to validate our overall tool and not individual components. Last but not least, although we took every effort to make the simulation as high fidelity as possible, this rubric has only been used to evaluate simulated resuscitation performance, and may not entirely reflect provider actions in an actual clinical environment.

## Conclusion

A standardized video assessment tool has potential to provide a valid and reliable method to assess 12 performances of clinicians facing simulated medical emergencies. This will serve as a standard measurement instrument to assess the efficacy of novel decision aids and care models, such as CERTAIN, on clinical performance before their implementation at the bedside at our institution.

## Additional files

Additional file 1:
**Appendix 1.** Standardized Clinical Scenario Script. **Appendix 2.** Complete Scoring Definitions. (DOCX 29 kb) Additional file 2: Electronic Supplement: Additional tables. (DOCX 22 kb)

## Abbreviations

ACLS: advanced cardiac life support
ACS: acute coronary syndrome
CERTAIN: Checklist for Early Recognition and Treatment of Acute IllNess
ICU: intensive care unit
