# Supplementary material for: Development and validation of clinical performance assessment in simulated medical emergencies: an observational study
Source: BMC Emerg Med. 2016 Jan 15;16:4. doi: 10.1186/s12873-015-0066-x (PMC4715281; doi:10.1186/s12873-015-0066-x)
Supplement: Additional file 1: — Appendix 1. Standardized Clinical Scenario Script. Appendix 2. Complete Scoring Definitions. (DOCX 29 kb) [file 12873_2015_66_MOESM1_ESM.docx]

# Appendix 1: Standardized Clinical Scenario Script

Clinical Vignette: Low Blood Pressure (Sepsis) in the Floor

A 70 year old male comes from a nursing home with generalized weakness, malaise and loss of appetite. He arrived to the floor as direct admission 10 min ago. He received 1L of normal saline and oxygen 2L by EMS during transport for Low Blood Pressure. You are the admitting doctor on the medicine floor and you have not seen the patient before.

**Code status:** Full Code

**PMHX:** Polymyalgia rheumatica, Diabetes Mellitus Type 2 and mild Chronic Heart Failure (CHF)

**Medications:** Prednisone 10mg, Insulin, Metoprolol and Atorvastatin.

**Allergies:** No Known Drug Allergies

**Personnel:** 2 Bedside nurses: principal nurse and auxiliary nurse.

Clinical Course

|  | State 1  (Start) | Stage 2  4 min | Stage 3  8 min |
| --- | --- | --- | --- |
| Symptoms | Generalized weakness, malaise and loss of appetite | Somnolent and poor responsive | Transfer to ICU |
|  |  |  |  |
| Physical Examination | AOX3, tachypneic. CV: No JVP, NL S1 and S2 tachycardia, no murmurs.  Lungs bilaterally clear,  Abdomen NL  Back: flank tenderness bilateral  CNS no new focal | AO, Tachypneic  Mottle skin  Decreased capillary refill  Glasgow 7 | Unchanged |
| Temperature | 38.4 | 39 | Improving vs Code* |
| Heart Rate | 106 | 128 | Improving vs Code* |
| Blood Pressure | 89/50 | 77/40 | Improving vs Code* |
| Respiratory Rate | 22 | 28 | Improving vs Code* |
| Oxygen Saturation | 98% on 2 L | 94% on 2L | Improving vs Code* |
| Cardiac Rhythm | Sinus Tachycardia | Sinus Tachycardia | Improving vs Code* |
| Vascular Access | None | Peripheral IV or central line if requested | Peripheral IV or central line if requested |
| ABCDE | A: Normal  B: Normal  C: Mottling  D: Awake  E: Normal | A: Normal  B: Normal  C: Mottling and weak pulse  D: P  E: NL | Improving vs Code* |
| Laboratory | ------ | WBC 15000, Hematocrit 23, platelets 95000/mm3, Serum Creatinine 2.1, Sodium 130, potassium 3.5, Bicarbonate 16  Lactate 4.6, glucose 45  ABG: pH: 7.28 O2:122 CO2 14  HCO3: 12 Sat 88%  UA if asked for it – WBC>100, Leuko esterase +++ |  |
| Test Result | ------ | CXR normal  CC ultrasound: Normal LV, RV, IVC <2 cm, >50% collapsible  right hydronephrosis |  |
| Intervention | Oxygen NC  IV access  IV fluids | Fluid Bolus  Vasopressors  Central Line  Antibiotics  Cultures  Stress dose steroids  Glucose administration | Final Recommendations |

*Decision to have patient improve versus code was based on completion of a single key task in that scenario:

Case 1 hypotension 🡪 Start Vasopressors

Case 2 hypoxia 🡪 Recognize the need of invasives mechanic ventilation

 Case 2 chest pain 🡪 Electrical cardioversion on patient with unstable tachyarrhythmia

# Appendix 2: Complete Scoring Definitions

**General Items For all cases:**

| **Items** | **Provider Actions which count as “Done”** | **Provider Actions which do NOT count as “Done”** |
| --- | --- | --- |
| Assess Code Status | Any discussion with the patient or nurse about whether the patient wants CPR and/or intubation |  |
| Evaluate “A” | Assessment of As (explicitly any of the following): Airway compromise, stridor, wheezing; Alternatively may say something like: “apparently free airway”, etc | Just talking to the patient without verbalizing that this indicates a free airway |
| Evaluate “B” | Assessment of Bs (explicitly any of the following): Poor air entry, Crackles, Work of Breathing; Alternatively may say something like: “apparently no breathing problems”, or ask for/do lung auscultation etc. | Just talking to the patient without verbalizing that this indicates normal breathing function |
| Evaluate “C” | Assessment of Cs (explicitly any of the following): ECG monitor, pulse status, mottling; Alternatively may ask for “cardiac monitoring”, or say something like: “apparently intact circulation”, etc | Just asking for vitals (monitor) in general without indicating specifically cardiac monitoring |
| Evaluate “D” | Assessment of Ds (explicitly any of the following): Level of consciousness (AVPU), seizures, focal deficits; Alternatively may say something like: “apparently awake and oriented/ unresponsive”, etc | Just talking to the patient without verbalizing that this indicates normal level of consciousness |
| Evaluate “E” | Assessment or asking for Es (explicitly any of the following): Abdominal distension, overt bleeding, skin abnormalities. | Just looking at dummy (even if removing gown or blanket) without verbalizing the reason behind |
| Check Vitals Sign | Asking for vital signs | Asking for temperature only |
| Check Temperature | Asking for temperature; Alternatively may ask for fever or if the patient feels hot/cold etc | Asking only for vitals in general |
| Review Past Medical History | Asking for past medical history/ previous diagnoses to the dummy or nursing personal | Just re-stating the diagnoses mentioned by researchers in the standard introductory text for the case scenario |
| Review Medications | Asking for home medications to the dummy or nursing personal |  |
| Review Allergies | Asking for known drug allergies to the dummy or nursing personal |  |
| Order Labs | Ordering any lab tests (including point-of-care labs) |  |
| Order Oxygen | Considering the need of or ordering supplemental oxygen (any FiO2, any device) |  |
| Review Differential Diagnoses | Considers at least one alternative diagnosis different from the (apparent) working hypothesis | Considering and treating according to only one diagnosis |

**Specific items for case-1 scenarios:**

| **Items** | **Provider Actions which count as “Done”** | **Provider Actions which do NOT count as “Done”** |
| --- | --- | --- |
| **Cues** |  |  |
| C -> Find Mottling | Finding out that patient has skin mottling |  |
| Recognize Hypoglycemia | Realizing that patient has low glucose/ “blood sugar”/ hypoglycemia | Just reading out/ re-stating the raw lab values |
| **Diagnostics** |  |  |
| Any Cultures | Considering or ordering any cultures or pan-cultures |  |
| -Blood Cultures^1^ | Considering or ordering blood cultures |  |
| -Urine Cultures^1^ | Considering or ordering urinary cultures |  |
| -Respiratory Cultures^1^ | Considering or ordering respiratory cultures (sputum, tracheal secretions, BAL) |  |
| CXR | Considering or ordering a chest x-ray or CT |  |
| Bedside US | Considering or ordering an Echo/ (bedside) ultrasound/ FAST, critical-care US (CCUS)/ RUSH-exam |  |
| **Medications and other Treatments** |  |  |
| Give Fluid Bolus | Considering or ordering at least 1 liter per 1 hour (or at least 250ml at the same rate or faster, e.g. 500ml over 30 min or less); if the time frame is not specified the provider needs to indicate at least that the fluids should be given fast (e.g. “run wide open”, “put pressure cuff around the saline bag”, etc) | Just ordering “some fluids”, “100ml/h”, “give 1 liter” (without any other comments), etc. |
| Give Vasopressor | Considering or ordering any amount or type of vasopressor (including: “give vasopressors”) | Just mentioning that blood pressure should be increased |
| - Central line | Considering or ordering a central line (e.g “IJ”, “subclavian catheter”, etc.) |  |
| Give Stress Dose Steroids | Considering or ordering any amount or type of glucosteroids (including: “give stress dose steroids”) | Just mentioning “steroids” (e.g. for COPD rather than hemodynamic stabilization). |
| Give Antibiotics | Considering or ordering any amount or type of antibiotic (including: “give antibiotics”) |  |
| Source Control | Considering or ordering an exam (e.g. US/CT) with the explicit goal to identify and fix possible infection focus/ foci (including “remove/change of devices”) Also: contacting Interventional Radiology, urology, surgery consult for wound/abscess/ hydronephrosis drainage | Just mentioning “source control” or test without any further specifications (e.g. “get ultrasound”) |
| Give IV Glucose for Hypoglycemia | Considering or ordering Glucose/ Dextrose/ Amp D50/ Glucagon or sugary drink/food (e.g. orange juice, cola, cookie) |  |
| **Mechanical Ventilation** |  |  |
| - Prepare materials for intubation | Preparing/Instructing to prepare specific intubation materials including at least 3 of the following: laryngoscope, tube, suction, end tidal CO2, ambu-bag/anesthesia mask | Just pulling/instructing to get the intubation cart |
| - Preoxygenate | Instructing or performing preoxygenation (e.g. using ambu-bag, “give 100% oxygen”, “lets fully saturate”, etc). BiPAP can be count as pre-oxygenation if the provider specifies this out loud. | Just leaving supplemental oxygen devices on till intubation |
| - Give Sedation | Considering or ordering any amount or type of sedation (including: “give sedation”) |  |
| - Intubate | Performing/ Ordering intubation (even if it done by another team member) |  |
| - Specify Ventilation Mode | Stating explicitly the desired ventilation mode at least 2 of the following: Mode (AC, SIMV, PS) Tidal Volume, Respiratory Rate, PEEP and FiO2. | Just connecting to/ ordering mechanical ventilation |
| - Order Lung Protective Ventilation/ Volume | Considering or ordering lung protective ventilation (e.g. ordering “low tidal volume” or a tidal volume of no more than 8ml/kg predicted body weight (approximately) |  |

**Specific items for case-2 scenarios:**

| **Items** | **Provider Actions which count as “Done”** | **Provider Actions which do NOT count as “Done”** |
| --- | --- | --- |
| **Cues** |  |  |
| A -> Find Wheezing | Finding out that patient has wheezing |  |
| B -> Find Crackles | Finding out that patient has crackles |  |
| C -> Find Sinus Tachycardia | Realizing that patient develops tachycardia (bpm >100); it is not necessary to identify definitely that it is a sinus tachycardia |  |
| E -> Find Low Urinary Output | Finding out that patient has low urinary output or asking about urinary output in general |  |
| **Diagnostics** |  |  |
| Any cultures | Considering or ordering any cultures (alternatively: “pan-cultures”) |  |
| -Blood Cultures^1^ | Considering or ordering blood cultures |  |
| -Urine Cultures^1^ | Considering or ordering urinary cultures |  |
| -Respiratory Cultures^1^ | Considering or ordering respiratory cultures |  |
| Nasal Swab | Considering or ordering nasal swabs to test for influenza or RSV |  |
| Serology | Considering or ordering serology specific for pneumonia eg. Pneumococcal antigen |  |
| Urinary Antigens | Considering or ordering urinary antigen specific for pneumonia, e.g. Legionella antigen |  |
| CXR | Considering or ordering a chest x-ray or CT |  |
| Bedside US | Considering or ordering an Echo/ (bedside) ultrasound/ FAST, critical-care US (CCUS)/ RUSH-exam |  |
| **Medications and other Treatments** |  |  |
| Give Fluid Bolus | Considering or ordering at least 1 liter per 1 hour (or at least 250ml at the same rate or faster, e.g. 500ml over 30 min or less); if the time frame is not specified the provider needs to indicate at least that the fluids should be given fast (e.g. “run wide open”, “put pressure cuff around saline bag”, etc) | Just ordering “some fluids”, “100ml/h”, “give 1 liter” (without any other comments), etc. |
| Give Antibiotics | Considering or ordering any amount or type of antibiotic (including: “give antibiotics”) |  |
| **Mechanical Ventilation** |  |  |
| - Prepare materials for intubation | Preparing/Instructing to prepare specific intubation materials including at least 3 of the following: laryngoscope, tube, suction, end tidal CO2, ambu-bag/anesthesia mask | Just pulling/instructing to get the intubation cart |
| - Preoxygenate | Instructing or performing preoxygenation (e.g. using ambu-bag, “give 100% oxygen”, “lets fully saturate”, etc). BiPAP can be count as pre-oxygenation if the provider specifies this out loud. | Just leaving supplemental oxygen devices on till intubation |
| - Give Sedation | Considering or ordering any amount or type of sedation (including: “give sedation”) |  |
| - Intubate | Performing/ Ordering intubation (even if it done by another team member) |  |
| - Specify Ventilation Mode | Stating explicitly the desired ventilation mode at least 2 of the following: Mode (AC, SIMV, PS) Tidal Volume, Respiratory Rate, PEEP and FiO2. | Just connecting to/ ordering mechanical ventilation |
| - Order Lung Protective Ventilation/ Volume | Considering or ordering lung protective ventilation (e.g. ordering “low tidal volume” or a tidal volume of no more than 8ml/kg predicted body weight (approximately) |  |

**Specific items for case-3 scenarios:**

| **Items** | **Provider Actions which count as “Done”** | **Provider Actions which do NOT count as “Done”** |
| --- | --- | --- |
| **Diagnostics I** |  |  |
| Order FIRST EKG | Considering or ordering a first EKG |  |
| Order Cardiology Consult | Considering or ordering a cardiology consult (alternatively: calling STEMI pager, activating STEMI protocol, or contacting cath lab emergently etc) |  |
| CXR | Considering or ordering a chest x-ray or CT |  |
| Bedside US | Considering or ordering an Echo/ (bedside) ultrasound/ FAST, critical-care US (CCUS)/ RUSH-exam |  |
| **Medications and other Treatments I** |  |  |
| - Aspirin | Considering or ordering any dose of Aspirin |  |
| - Second Anti-Platelet Therapy | Considering or ordering any dose or type of second anti-platelet therapy (e.g. clopidrogel/ “Plavix^®^” ticagrelor, abciximab/Reapro, eptifibatide/integrillin, tirofiban/aggrastat) |  |
| - Analgesia | Considering or ordering any dose or type of analgesia (including “give something for pain”) |  |
| - Nitroglycerin | Considering or ordering any dose or type of nitroglycerin |  |
| - PCI or Thrombolysis | (Considering) contacting cath lab (alternatively may say: “call STEMI pager”, “activate STEMI protocol”, “call the cardiology fellow stat”) or considering thromblysis (any dose/type including “give thrombolysis”) | Considering or ordering a cardiology consult only |
| **Diagnostics II** |  |  |
| Order SECOND EKG | Considering or ordering a second EKG (after patient’s heart rhythm changed to A-fib) |  |
| Recognize V Tach | Realizing that patient has V-Tach (alternatively: “non-perfusing rhythm”, “is pulseless”, “coding”, “needs CPR”, “V-fib”, etc) |  |
| **Medications and other Treatments II** |  |  |
| Cardioversion | Considering or ordering electrical cardioversion | Considering or ordering medical/pharmacological cardioversion only |
| - Give Sedation | Considering or ordering any dose or type of sedation (including “give sedation”) |  |
| - Specify Mode of Cardioversion | Setting/ Instructing a specific cardioversion mode (mono- vs biphasic) |  |
| - Specify Energy Level | Setting/ Instructing a specific energy level in Joules |  |
