# Supplementary material for: Development and validation of clinical performance assessment in simulated medical emergencies: an observational study
Source: BMC Emerg Med. 2016 Jan 15;16:4. doi: 10.1186/s12873-015-0066-x (PMC4715281; doi:10.1186/s12873-015-0066-x)
Supplement: Additional file 2: — Electronic Supplement: Additional tables. (DOCX 22 kb) [file 12873_2015_66_MOESM2_ESM.docx]

# Electronic Supplement: Additional tables

## Table E1- Case 1 Checklist items. Items with N/A were uniformly not performed, and thus timing agreement cannot be calculated. Items marked with * were below our threshold for inclusion in the final rubric. **Note that only one subject was categorized as novice for case 1, and thus all items were either 100% or 0%.

| Item | % Overall Agreement | % agreement on timing (within 15 seconds | Done by experts  (N=4) | Done by novice**  (N=1) |
| --- | --- | --- | --- | --- |
| Identified skin mottling? | 80% | 100% | 0 (0%) | 1 (100%) |
| Bolused IV fluids?* | 100% | 20% | 4 (100%) | 1 (100%) |
| Obtained cultures (any type) | 80% | 100% | 4 (100%) | 1 (100%) |
| Obtained blood cultures? | 100% | 100% | 3 (75%) | 1 (100%) |
| Obtained urine cultures? | 60% | 100% | 2 (50%) | 0 (0%) |
| Obtained respiratory cultures? | 80% | N/A | 4 (100%) | 1 (100%) |
| Administered stress dose steroids? | 100% | 100% | 1 (25%) | 0 (0%) |
| Identified hypoglycemia? | 80% | 100% | 2 (50%) | 0 (0%) |
| Administered IV glucose? | 60% | N/A | 2 (50%) | 0 (0%) |
| Antibiotics given? | 100% | 60% | 4 (100%) | 1 (100%) |
| Vasopressors initiated?* | 80% | 25% | 4 (100%) | 1 (100%) |
| Central line placed? | 80% | 100% | 4 (100%) | 1 (100%) |
| Ultrasonography performed? | 80% | N/A | 4 (100%) | 1 (100%) |
| Chest X ray obtained? | 80% | 100% | 3 (75%) | 0 (0%) |
| Source of infection addressed? | 100% | N/A | 0 (0%) | 0 (0%) |
| Patient intubated? | 80% | 100% | 2(50%) | 0 (0%) |
| Intubation preparation discussed?* | 80% | 50% | 3 (75%) | 0 (0%) |
| Preoxygenation performed? | 80% | 100% | 2 (50%) | 1 (100%) |
| Sedation performed | 60% | N/A | 1 (25%) | 0 (0%) |
| Ventilator mode ordered? | 100% | N/A | 0 (0%) | 0 (0%) |
| Ventilator set for lung protective ventilation? | 100% | N/A | 0 (0%) | 0 (0%) |
| **Pooled reliability:** | **83.8%** | **73.0%** | **58.3%** | **47.6%** |

## Table E2- Case 2 Checklist items. Items with N/A were uniformly not performed, and thus timing agreement cannot be calculated. Items marked with * were below our threshold for reliability and were not included in the final rubric.

| Item | % Overall Agreement | % Agreement in items which both reviewers classified as "Done" | Done by expert  (N= 4) | Done by novices  (N=4) |
| --- | --- | --- | --- | --- |
| Identified wheezing? | 86% | 100% | 3 (75%) | 3 (75%) |
| Identified crackles? | 86% | N/A | 3 (75%) | 2 (50%) |
| Identified sinus tachycardia?* | 29% | 0% | 4 (100%) | 1 (25%) |
| Identified low urine output | 100% | N/A | 0 (0%) | 0 (0%) |
| Administered fluid bolus* | 57% | 0% | 1 (25%) | 2 (50%) |
| Obtained cultures (any type) | 71% | 100% | 4 (100%) | 2 (50%) |
| Obtained blood cultures?* | 57% | 100% | 4 (100%) | 2 (50%) |
| Obtained urine cultures? | 71% | N/A | 1 (25%) | 0 (0%) |
| Obtained respiratory cultures? | 86% | N/A | 0 (0%) | 0 (0%) |
| Antibiotics administered?* | 57% | N/A | 3 (75%) | 2 (50%) |
| Ultrasonography performed? | 71% | N/A | 0 (0%) | 0 (0%) |
| Chest X ray obtained? | 71% | 100% | 4 (100%) | 3 (75%) |
| Influenza nasal swab obtained? | 100% | N/A | 0 (0%) | 0 (0%) |
| Respiratory serologies obtained? | 100% | N/A | 0 (0%) | 0 (0%) |
| Urinary antigen tests obtained? | 100% | N/A | 0 (0%) | 0 (0%) |
| Intubation preparation discussed? | 71% | 100% | 4 (100%) | 3 (75%) |
| Preoxygenation performed? | 71% | 100% | 4 (100%) | 3 (75%) |
| Sedation performed | 71% | 100% | 4 (100%) | 4 (100%) |
| Patient intubated? | 86% | 100% | 4 (100%) | 4 (100%) |
| Ventilator mode ordered? | 100% | N/A | 1 (25%) | 0 (0%) |
| Ventilator set for lung protective ventilation? | 100% | N/A | 1 (25%) | 0 (0%) |
| **Pooled reliability:** | **78.2%** | **81.8%** | **53.6%** | **36.9%** |

## **Table E3- Agreement between raters on Case 3 Checklist items. Items with N/A were uniformly not performed, and thus timing agreement cannot be calculated. Items marked with * were below our threshold for inclusion in the final rubric.**

| Item | % Overall Agreement | % Agreement in items which both reviewers classified as "Done" | Done by expert  (N=3 ) | Done by novices  (N=2) |
| --- | --- | --- | --- | --- |
| EKG obtained?* | 100% | 50% | 3 (100%) | 2 (100%) |
| Cardiology/Cath lab consulted?* | 75% | 50% | 3 (100%) | 1 (50%) |
| Aspirin given?* | 50% | 100% | 3 (100%) | 1 (50%) |
| Second antiplatelet given? | 100% | N/A | 0 (0%) | 0 (0%) |
| Analgesia administered?* | 25% | 100% | 3 (100%) | 2 (100%) |
| Nitroglycerin given? | 75% | 100% | 2 (67%) | 1 (50%) |
| Thrombolysis/PCI requested?* | 50% | 100% | 3 (100%) | 1 (50%) |
| Ultrasonography performed? | 100% | N/A | 0 (0%) | 0 (0%) |
| Chest X ray obtained? | 100% | 100% | 2 (67%) | 2 (100%) |
| EKG repeated with rhythm change? | 100% | 100% | 1 (33%) | 0 (0%) |
| Ventricular tachycardia identified?* | 50% | 100% | 1 (33%) | 0 (0%) |
| Cardioversion performed?* | 100% | 0% | 2 (67%) | 0 (0%) |
| Sedation performed?* | 100% | 0% | 2 (67%) | 0 (0%) |
| Mode of cardioversion specified? | 75% | N/A | 2 (67%) | 0 (0%) |
| Energy for cardioversion specified? | 100% | N/A | 0 (0%) | 0 (0%) |
| **Pooled reliability:** | **80.0%** | **72.2%** | **60.0%** | **33.3%** |
